# Selection of Target Nutrients for the Nutritional Standards of School Lunches in Korea

**DOI:** 10.3390/nu11112578

**Published:** 2019-10-25

**Authors:** Meeyoung Kim, Dongwoo Kim, Jihyun Yoon

**Affiliations:** 1 Department of Food and Nutrition, Seoul National University, Seoul 08826, Korea; mee1242@snu.ac.kr; 2 Department of Human Ecology, Korea National Open University, Seoul 03087, Korea; kimdow@knou.ac.kr; 3 Research Institute of Human Ecology, Seoul National University, Seoul 08826, Korea

**Keywords:** NHANES, foodservice, nutrition assessment, Dietary Reference Intakes, school lunch program

## Abstract

The purpose of this study was to select target nutrients to be included in the nutritional standards of school lunches in Korea. The dietary intake data for children and adolescents aged 6–17 years old from the Korea National Health and Nutrition Examination Survey VI were analyzed for eight groups based on gender and age (6–8, 9–11, 12–14, and 15–17 years old). First, the usual intake of 3091 subjects was estimated and assessed to identify nutrients with insufficient or excessive intake prevalence. Along with the nutrients identified by the assessment, the energy and nutrients prioritized in the meal planning procedure of the 2015 Dietary Reference Intakes for Koreans were the initial candidates; these nutrients and energy include the percentages of energy from carbohydrates, protein, and fat; vitamin A; riboflavin; niacin; vitamin C; calcium; phosphorus; sodium; and iron. Phosphorus was excluded as a result of there being little evidence of clinical symptoms caused by its insufficient intake. Sodium was excluded because reliable data on added salt were not available among the school lunch recipes in Korea. Therefore, energy; the percentages of energy from carbohydrates, protein, and fat; vitamin A; riboflavin; niacin; vitamin C; calcium; and iron were selected to be included in the nutritional standards for school lunches in Korea.

## 1. Introduction

School lunches play a key role in school students’ health in South Korea (Korea hereafter), because most students are participating in school lunch programs. As of February 2019, 100% of the 11,818 elementary, middle, high, and special need schools operated school lunch programs, and 99.9% of the 5,612,000 students attending these schools participated in the programs in Korea [1]. Although school lunches are available in all the schools, school breakfasts and dinners are served by a few boarding schools and many high schools, respectively.

Students are provided free or paid school lunches in Korea. As of March 2019, 89.5% of all the students were provided free school lunches; all the students in all the elementary, middle, and special need schools are provided free school lunches, as well as in some high schools, whereas only the students from low-income families eat free school lunches in the remaining high schools [2]. On the other hand, most of the school lunch programs (98%) are self-operated [1]. Either nutrition teachers or school dietitians are in charge of the school lunch programs; a nutrition teacher has a teacher license in addition to a dietitian license.

School lunches should provide the required nutrients to promote the development, growth, and health of students. The nutritional standards of school lunches can be an important tool to ensure that proper nutrients are parts of children’s and adolescents’ diets. Several studies have shown that the nutritional quality of school meals using nutritional standards was higher than that of school meals prepared without those standards [3,4,5]. 

Korea has nutritional standards for lunches, but not for breakfasts and dinners. The nutritional standards of school lunches in Korea are mandatory for all the schools operating school lunch programs. In Korea, the nutritional standards of school lunches were first established in the School Meals Act of 1981. Since then, the standards have been revised several times in 1993, 1997, and 2007. Currently, the 2007 standards based on the 2005 Dietary Reference Intakes for Koreans (KDRIs) [6] are being used, although the KDRIs were revised twice in 2010 and 2015. For this reason, school lunch experts have highlighted the urgent need to update the nutritional standards of school lunches in Korea [7].

Worldwide, the nutritional standards of school lunches are nutrient-based, food-based, or both, depending on the country [8]. In Korea, these standards have been nutrient-based since the first version. The nutrient-based standards of school lunches have usually been developed in two steps: (1) selecting the target nutrients to be included in the standards and (2) establishing the reference values of the target nutrients for school lunches. Both steps must use scientific methods. For the first step, it is important to assess the most recent dietary intake of students and reflect the results in the nutrient selection procedure.

The nutrients included in the nutritional standards of school lunches vary from country to country. For example, along with energy, nutrients included in the current standards are different for Korea (9 nutrients), Taiwan (4 nutrients), and Japan (12 nutrients). For macronutrients, protein and fat are both included in the standards of all three countries. All three countries also include calcium in their school lunch standards. Vitamin A, thiamin, riboflavin, and vitamin C are included in Korea and Japan, but no vitamins are included in Taiwan [8]. 

The nutrients included in the nutritional standards of school lunches have changed over time in Korea. The first nutritional standards of school lunches in 1981 included energy, protein, vitamin A, thiamin, riboflavin, niacin, vitamin C, calcium, and iron [9]. Vitamin D was added in the revised standards of 1993 [10]. In the revised standards of 2007, niacin and vitamin D were excluded and the percentages of energy from carbohydrates, protein, and fat were added. The current standards established in 2007 include reference values for energy; percentages of energy from carbohydrates, protein, and fat; protein; vitamin A; thiamin; riboflavin; vitamin C; calcium; and iron [11]. The reference values were one-third of the recommended daily intake for each selected nutrient [8].

It is imperative to revise these standards because they are more than 10 years old. Science-based standards should be developed, reflecting the current dietary intake of Korean students, as the basis for this revision. The purpose of this study was to select the target nutrients to be included in the new nutritional standards of school lunches in Korea, as the first step in the developmental procedure of nutrient-based nutritional standards.

## 2. Subjects and Methods 

We first estimated and assessed the usual dietary intakes of Korean school aged-children and adolescents. The initial candidates for the target nutrients were selected based on the assessment results. In addition, the high priority nutrients in the meal planning procedure of the 2015 KDRIs were included as initial candidates. Then, we excluded some nutrients from the initial candidates. Nutrients that have little evidence of clinical symptoms if they are insufficient in one’s diet, or those that were difficult to apply in the current school lunch programs in Korea owing to the absence of reliable data, were excluded. A detailed description of the usual intake estimation and dietary assessment to select the initial candidates for inclusion in the standards is presented below. 

### 2.1. Data Source and Subjects

The dietary data used in this study were obtained from one-day, 24 h recall data in the Korea National Health and Nutrition Examination Survey VI (KNHANES VI). This survey was conducted for three years in 2013, 2014, and 2015. The 2013 and 2014 surveys were approved by the Institutional Review Board (IRB) of the Centers for Disease Control and Prevention in Korea (IRB approval number: 2013-07CON-03-4C, 2013-12EXP-03-5C). The 2015 survey was exempted from IRB review under the provisions of the Bioethics and Safety Act [12]. 

The KNHANES is a nationwide statutory investigation that is performed to understand the health and nutritional levels of Koreans and to establish and evaluate national health policies. Currently, the survey is conducted annually with approximately 10,000 Korean representatives. The survey uses stratified and multistage sampling, and consists of three parts: a health interview survey, a health examination survey, and a nutrition survey. The sample population for the survey is non-institutionalized Korean civilians aged one year or older [13].

The health interview and health examination surveys are performed at mobile examination centers that travel to survey areas, and the nutrition survey, including a one-day, 24 h recall for each respondent, was administered in respondents’ homes by trained interviewers consisting of two nutritionists. The nutrition survey using one-day, 24 h recall method included face-to-face interviews of the sampled persons. Intake aids such as 2D model collection for food and food containers, measuring cups, measuring spoons, 30 cm ruler, thickness ruler, and tape measure were used to investigate the dietary intake [14].

In this study, the dietary data of 3091 children and adolescents aged 6–17 years old (i.e., the educational statistical age group for elementary, middle, and high school students in Korea) among the dietary data of the KNHANES VI were used for analysis. The nutrient intake from dietary supplements was excluded from the analysis. The dietary assessment was conducted separately among the eight groups based on gender (male and female) and four age groups (6–8, 9–11, 12–14, and 15–17 years).

### 2.2. Estimation of the Usual Intake among School-Aged Children and Adolescents

Many Korean studies have used one-day, 24 h recall data to assess dietary intake. However, the nutrient intake for one day may be very different from an individual’s usual intake, which is the average long-term dietary intake, as there is considerable day-to-day variation [15]. Thus, researchers use a statistical methodology to adjust day-to-day variations [16,17,18] or use average multiple 24 h recalls.

Several statistical methods to estimate the usual intake have been developed over the last three decades. To apply these statistical methods to the adjustment of the nutrient intake, however, it is necessary to have at least two-day, 24 h recalls. Because the KNHANES used one-day, 24 h recall data, another methodology was needed. The details of the methodology, using external variance components through the Iowa State University (ISU) method [19], have been discussed in detail elsewhere [20]. Several studies estimated the usual intake using the method that input the within-individual variance components derived from the external data [20,21] or from the data of some subjects [22,23] through the ISU method when only one-day, 24 h recall data were collected.

In our study, the external within-individual variance components estimates from the 2007–2009 Dietary Intake Survey of Infants, Children, and Adolescents [24], which was conducted by the Korea Health Industry Development Institute (KHIDI) (two-day recall), were used to adjust the day-to-day variations of one-day, 24 h recall data from the KNHANES VI. The usual intake of energy and 13 nutrients, of which the within-individual variances could be calculated, was estimated among energy and 45 nutrients included in one-day, 24 h recall data from the KNHANES VI. The usual intake of energy, protein, vitamin A, thiamine, riboflavin, niacin, vitamin C, calcium, phosphorous, sodium, potassium, and iron was estimated. The usual intake of the percentages of energy from carbohydrates, protein, and fat was also estimated. The mean; standard deviation (SD); and 5th, 10th, 25th, 50th, 75th, 90th, and 95th percentiles of the usual intake were analyzed. 

### 2.3. Assessment of the Dietary Intake among School-Aged Children and Adolescents

The dietary intake was assessed for eight groups among the subjects using the estimated usual intake data. The insufficient and excessive intake prevalence of the subjects for energy as well as the percentages of energy from carbohydrates, protein, and fat were assessed. When the estimated average requirements (EARs) had been set for certain nutrients such as protein, vitamin A, thiamine, riboflavin, niacin, vitamin C, calcium, phosphorous, and iron, the prevalence of insufficient intake was assessed. In addition, the excessive intake prevalence was assessed for vitamin A, niacin, vitamin C, calcium, phosphorous, sodium, and iron; these nutrients had a tolerable upper intake level (UL) or an intake goal. 

#### 2.3.1. Energy and the Percentages of Energy from Carbohydrates, Protein, and Fat 

For energy, the percentages of average energy intake to meet estimated energy requirements (EERs) were calculated according to group. The proportions of the subjects below and above the acceptable macronutrient distribution ranges (AMDRs) were calculated by group for carbohydrates, protein, and fat. The AMDRs were 55%–65% for carbohydrates, 7%–20% for protein, and 15%–30% for fat for children and adolescents aged 6–17 years [25].

#### 2.3.2. Nutrients

For assessment of insufficient intake prevalence of nutrients by group, the EAR cut-point method [17] was used to calculate the proportion of the subjects below the EAR. However, for females aged 12–17 years old, it would not be appropriate to assess insufficient iron intake using the EAR cut-point method because the distribution of iron requirements is asymmetric around the EAR because of menstrual blood [17]. Therefore, the probabilities of inadequate iron intake for females aged 12–14 and 15–17 years old were calculated using the full probability approach [26]. The bioavailability of iron in a usual meal among Koreans is 12% [25]. However, the intake ranges for the probability of inadequate iron intake among females aged 12–14 and 15–17 years old with 12% bioavailability were not found in the literature. Thus, when using the full probability approach in this study, the intake ranges for the probability of inadequate iron intake of females aged 11–13 and 14–18 years old [27] were applied to females aged 12–14 and 15–17 years old, respectively. 

To calculate the total probability of inadequate iron intake for females aged 12–17 years old, we analyzed the proportion of females in the intake range for each probability of inadequacy. Then, the prevalence of inadequacy according to the intake range was calculated by multiplying the proportion of females corresponding to each intake range by the probability of inadequacy. Finally, the total probability of inadequate iron intake was obtained by adding up the prevalence of inadequacy within the range of each intake.

To assess the prevalence of the excessive intake of some nutrients, the proportion of the subjects above the UL was calculated by group. Although sodium did not have a UL because of the lack of evidence for setting it, the intake goal was set because of health risks from excessive intake [25]. Therefore, the proportion of the subjects above the intake goal for sodium was calculated by group; the proportions were calculated only for males and females aged 9–17 years, because the intake goal was not set for males and females aged 6–8 years in the 2015 KDRIs [25].

### 2.4. Statistical Analysis 

To estimate usual intake using the ISU method, we used Software for Intake Distribution Estimation (PC-SIDE) (version 1.0. Department of Statistics in Iowa State University, Ames, IA, U.S.). The average energy intake and the proportions of the subjects with insufficient and excessive intake were analyzed using SPSS (version 22. SPSS Inc., Chicago, IL, U.S.). For the inadequacy probability of iron intake for females aged 12–17 years old, we used Excel spreadsheets (version 2013. Microsoft, Raymond, Washington, DC, U.S.).

## 3. Results

### 3.1. Usual Intake of School-Aged Children and Adolescents

The within-individual variance components estimated through the ISU method using the two-day, 24 h recall data from the 2007–2009 Dietary Intake Survey of Infants, Children, and Adolescents [24] are shown in Table 1. The usual intake using these within-individual variance components for energy and 11 kinds of nutrients (protein, vitamin A, thiamin, riboflavin, niacin, vitamin C, calcium, phosphorus, sodium, potassium, and iron) and the usual intake of the percentages of energy from three kinds of nutrients (carbohydrates, protein, and fat) were estimated for Korean school-aged children and adolescents and are presented in Tables S1–S6.

### 3.2. Dietary Intake Status of School-Aged Children and Adolescents

The findings show that Korean school-aged children and adolescents had insufficient intake of vitamin A, riboflavin, niacin, vitamin C, calcium, phosphorus, and iron, and excessive intake of the percentage of energy from carbohydrates and sodium.

#### 3.2.1. Energy and the Percentages of Energy from Carbohydrates, Protein, and Fat 

Table 2 shows the percentage of the average usual energy intake to the EERs among Korean school-aged children and adolescents. School-aged children and adolescents consumed energy in the range of 96.0%–112.5% of their EER, depending on the group. Therefore, their energy intake was generally proper across gender and age groups.

Table 3 presents the proportions of Korean school-aged children and adolescents with insufficient and excessive intake of the percentages of energy from carbohydrates, protein, and fat. The proportions of the subjects below the AMDRs were 1.6%–19.3% for carbohydrates, 0.0% for protein, and 0.0%–1.9% for fat by group. The proportions of the subjects above the AMDRs were 11.2%–36.6% for carbohydrates, 0.0%–0.9% for protein, and 0.8%–12.8% for fat by group, indicating that the proportions of the subjects above the AMDR for carbohydrates were much higher than those for protein and fat.

#### 3.2.2. Nutrients

Table 4 and Table 5 present the proportion of Korean school-aged children and adolescents with insufficient and excessive nutrient intake, respectively. Table 6 shows the probability of inadequate iron intake for Korean females aged 12–17 years old, calculated using the full probability approach. Vitamin A, riboflavin, vitamin C, calcium, and phosphorus intake was not sufficient for school-aged children and adolescents. The proportions of the subjects below the EARs were relatively higher than for the other nutrients. In particular, the proportions of the subjects below the EARs for calcium were very high, ranging from 64.2% to 90.3% depending on the group. In addition, the proportions of the subjects below the EARs for vitamin A, riboflavin, vitamin C, and phosphorus were 41.8%–84.5%, 3.1%–38.4%, 21.3%–62.6%, and 0.2%–60.6%, respectively, by group.

For niacin and iron, the average proportions of insufficient intake for all subjects were as low as 13.4% and 14.1%, respectively. However, for females aged 12–14 and 15–17 years old, the proportions with insufficient intake were very high compared with the other groups. The proportions of the subjects below the EARs for niacin were 28.7% and 30.2% for females aged 12–14 and 15–17 years old, respectively. The probabilities of inadequate intake for iron were 37.8% and 41.1% for females aged 12–14 and 15–17 years old, respectively. Therefore, niacin and iron are of interest, especially for females aged 12–14 and 15–17 years old. For sodium, the proportions of children and adolescents aged 9–17 years old above the intake goal were very high, ranging from 81.4% to 98.3% by group.

### 3.3. Target Nutrients to Be Included in the Standards

**Figure 1:**
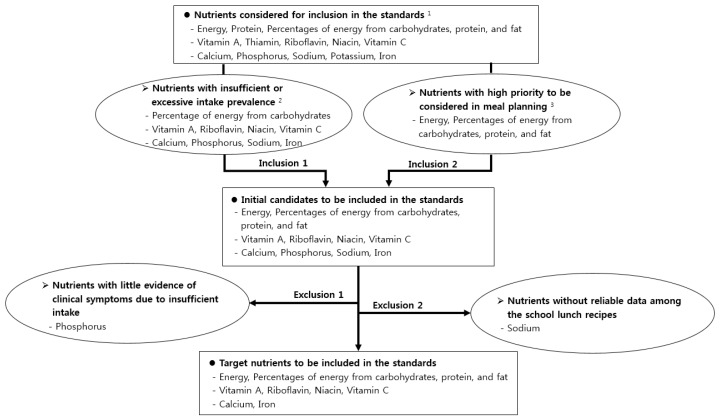
The selection procedure for target nutrients to be included in the nutritional standards of school lunches in Korea.^1^ Nutrients of which the usual intake was estimated in this study. ^2^ Nutrients with insufficient or excessive intake by school-aged children and adolescents according to the results of this study. ^3^ Nutrients considered first when planning meals in the 2015 Dietary Reference Intakes for Koreans.

The selection procedure for the target nutrients to be included in the nutritional standards of school lunches in Korea is shown in Figure 1.

#### 3.3.1. Initial Candidates to be Included in the Nutritional Standards

Initially, the percentage of energy from carbohydrates, vitamin A, riboflavin, niacin, vitamin C, calcium, phosphorus, sodium, and iron were selected as candidates. The dietary intake of such nutrients was found to be either insufficient or excessive among Korean school-aged children and adolescents. The 2015 KDRIs suggest that energy and the percentages of energy from carbohydrates, protein, and fat should be prioritized in the meal planning procedure [25]. Thus, they were also selected as initial candidates for inclusion. Therefore, we selected energy, the percentages of energy from carbohydrates, protein, and fat; vitamin A; riboflavin; niacin; vitamin C; calcium; phosphorus; sodium; and iron as initial candidates.

#### 3.3.2. Target Nutrients to Be Included in the Nutritional Standards

The next step included finalizing the list of target nutrients. Phosphorus was excluded because there was little evidence of clinical symptoms caused by insufficient intake [25,28]. Sodium was excluded because reliable data on added salt were not available among the school lunch recipes included in the National Education Information System (NEIS) in Korea. The NEIS is the official system used for nutrient analysis by dietitians and nutrition teachers in schools in Korea.

Finally, energy; the percentages of energy from carbohydrates, protein, and fat; vitamin A; riboflavin; niacin; vitamin C; calcium; and iron were selected as the target nutrients to be included in the nutritional standards of school lunches in Korea.

## 4. Discussion

Energy is necessary to maintain life and well-being. The body maintains a balance between intake and expenditure of energy. Individuals who consume more energy than they expend will gain weight. Individuals who expend more energy than they consume will lose weight. In other words, excessive intake of energy can cause obesity, which is a risk factor for various diseases [25]. In addition to the energy amount itself, the percentages of an imbalanced intake of energy from carbohydrates, protein, and fat can also increase the risk of several chronic diseases [29]. These are the components with priority in the meal planning procedure suggested in the 2015 KDRIs [25]. Therefore, it is appropriate for energy and the percentages of energy from carbohydrates, protein, and fat to be included in the nutritional standards of school lunches. 

In addition, this study found that the energy intake from carbohydrates was excessive among Korean school-aged children and adolescents. The upper limit of the AMDR for carbohydrates for children and adolescents aged 6–18 years old changed from 70% to 65% when the KDRIs were revised in 2015, because an excessive percentage of energy from carbohydrates, exceeding 70%, leads to health risks such as diabetes and metabolic syndrome [25]. Several studies have also reported that excessive carbohydrate intake is associated with a risk of metabolic syndrome [30,31,32]. Therefore, school lunches should be planned to ensure appropriate energy intake from carbohydrates among students.

The proportions of school-aged children and adolescents with an insufficient intake of vitamin A were high, ranging from 41.8% to 84.5% by group in this study. The proportion who consumed less than the EAR for vitamin A among Koreans was 45.3%, 44.0%, and 45.3% in 2013, 2014, and 2015, respectively [33]. However, in 2016 and 2017, the proportions were as high as 75.2% and 77.9%, respectively [33], because the unit of vitamin A changed from RE (retinol equivalent) with a ratio of 1/6 β-carotene to retinol equivalent to RAE (retinol activity equivalent) with a ratio of 1/12 β-carotene when the KDRIs were revised in 2015 [25]. The proportion of the population who consume less than the EAR for vitamin A in the RAE unit may be very high because vegetable foods with a high carotenoid content, such as carrots and spinach, are the main sources of vitamin A among Koreans [25]. 

On the other hand, there is concern that vitamin A intake might be underestimated or overestimated even after being adjusted for day-to-day variation because vitamin A tends to concentrate in a few animal foods containing retinol. In the case of Koreans, however, the major source of vitamin A is a variety of plant foods containing carotene [25]. Therefore, there is relatively less concern about underestimation or overestimation of vitamin A intake in the Korean population.

Riboflavin has been known as a nutrient with insufficient intake among Koreans in general. Previous studies also have reported that the intake of riboflavin was insufficient for middle and high school students in some regions of Korea [34,35]. 

The proportions of school-aged children and adolescents who consumed less than the EAR of vitamin C ranged from 21.3% to 62.6% by group. Sources of vitamin C are vegetables and fruits, and intake of these foods is closely associated with the prevention of cancer and cardiovascular disease [36,37,38]. However, it has been reported that the intake of vegetables and fruits is insufficient among school-aged children and adolescents in Korea. The proportions of children and adolescents aged 6–11 and 12–18 years old who consumed less than 500 g of vegetable and fruit per day, which is the goal of the 3rd National Health Promotion Plan, were 14.2% and 15.7%, respectively [33]. In addition, only 16.5% and 12.6% of middle and high school students, respectively, consumed vegetables more than three times per day [39]. Only 24.7% and 17.5% of middle and high school students, respectively, consumed fruits more than once per day [40]. Many studies have reported that Korean school-aged children and adolescents preferred vegetables less than other food ingredients [41,42,43,44].

For phosphorus, the proportions of subjects with insufficient intake ranged from 0.2% to 60.6% by group. The proportion of Koreans who consumed less than the EAR for phosphorus was 11.0% in 1998, increasing to 14.4% in 2015, 18.4% in 2016, and 17.6% in 2017 [33]. Phosphorus intake among Koreans was found to be particularly insufficient for 6–11, 12–18, and over 65 years old compared with other age groups [33]. However, it was reported that rare cases of deficiency symptom were observed in normal people [25]. For phosphorus, symptoms of deficiency are rare in normal individuals, although patients with chronic diseases or total parenteral nutrition tend to be deficient as the duration of their illness increases [45]. Thus, it is not necessary to manage phosphorus in the process of menu planning for school lunches. In fact, phosphorus is not included in the nutritional standards of school lunches in general.

For calcium, the proportion of school-aged children and adolescents with insufficient intake was the highest among the nutrients, ranging from 64.2% to 90.3% by group. According to the National Health Statistics (2017), the proportion of subjects with insufficient intake for calcium was also very high; these proportions were 70.0% and 73.7% for 6–11 year old males and females, respectively, and 73.7% and 85.8% for 12–18 year old males and females, respectively [33]. Abundant studies have also reported that calcium intake has been insufficient among Koreans [34,46,47]. 

The lack of calcium intake is related to the lack of milk intake. Korean school-aged children and adolescents consume milk inadequately, even though it is the top source of calcium among Koreans [33]. In fact, children and adolescents aged 6–11 and 12–18 years old consumed only 219 g and 172 g of milk per day, respectively [33]. The proportions of middle and high school students who consumed milk more than once per day were also very low, at 29.3% and 21.3%, respectively [39]. Many studies have shown that milk intake has a positive effect on bone density, physical growth, and physical strength among children and adolescents [48,49,50]. Therefore, calcium supplementation from consuming milk and dairy products or corresponding supplemental foods is urgently needed to promote calcium intake among children and adolescents during their growth periods [51,52]. Calcium is included in the school lunch standards of Japan and Taiwan [8], which have a similar food culture to Korea; consuming milk and dairy products during meals is not easy in all three countries.

Niacin and iron were the nutrients of interest because they showed a high prevalence of insufficiency for some groups. For niacin, the overall proportion of school-aged children and adolescents with insufficient intake was as low as 13.4% in this study. However, the proportions increased up to 28.7% and 30.2% for females aged 12–14 and 15–17 years old, respectively. The National Health Statistics (2017) reported a high prevalence of insufficient niacin intake for all the children and adolescents aged 6–18 years old; the proportions of subjects with insufficient intake for niacin were 25.2% and 38.5% for males and females aged 6–11 years old, respectively, and 35.9% and 56.2% for males and females aged 12–18 years old, respectively [33]. 

The overall proportion of the subjects with insufficient iron intake was as low as 14.1% in this study. However, the probability of inadequate iron intake was very high for females aged 12–14 (37.8%) and 15–17 years old (41.1%). Other studies have shown that Korean adolescents, particularly females, consumed iron insufficiently [34,46,47,53].

For sodium, the proportions of children and adolescents aged 9–17 years old with excessive sodium intake ranged from 81.4% to 98.3% by group. These are the levels of a health concern. According to the National Health Statistics (2017), the proportions of the children and adolescents aged 9–11 and 12–18 years old above the intake goal for sodium were also high, at 67.5% and 73.8%, respectively [33]. It was also reported that the ratio of the average intake of sodium to the sodium intake goal ranged from 1.6 to 1.8 times for middle school grades 1–2 [34] and 1.9 times for high school students [35]. Excessive sodium intake increases the incidence of cerebrovascular and cardiovascular diseases due to high blood pressure [54]. It has also been reported that excessive sodium intake has an influence on obesity, gastric cancer, urolithiasis, and osteoporosis [55,56,57]. 

Therefore, sodium is one of the most important nutrients to be managed when planning school lunch menus. However, sodium was excluded from the final target nutrients in this study because reliable data on added salt were not available among the school lunch recipes in Korea at the time of this study. Accurate recipe data including the added salt quantities should be entered into the NEIS as soon as possible. Sodium was included in the nutritional standards of school lunches in many countries such as the United States, Japan, and Taiwan [58,59,60]. In the United Kingdom (England), the nutritional standards of school lunches restricted sodium by limiting the intake of foods high in fat, sugar, and salt [61]. 

Vitamin A, riboflavin, vitamin C, calcium, and iron are the nutrients included in the nutritional standards of school lunches in Japan [8]. In Korea, they have been included in the nutritional standards of school lunches since the establishment of the standards in 1981. However, niacin was included in the standards only before 2007. Niacin was excluded from the revised standards in 2007 because sufficient protein intake leads to sufficient niacin intake [62]. In this study, however, niacin was selected to be included in the standards because it showed insufficient intake prevalence among some groups.

There are some limitations in this study. For nutritional quality, the ratio between individual fatty acids is more important than the total amount of fat. In addition, dietary fiber is crucial for diet quality. In this study, however, they could not be included for dietary intake assessment because the within-individual variances were not available for those nutrients. Recently, simple sugars have been underlined for their association with obesity and diabetes [63]. However, simple sugars could not be considered in this study from the beginning because the current nutrient database used for the KNHANES does not include the data of simple sugars. It is suggested that future studies on nutritional standards for school meals should consider such nutrients as fatty acids, dietary fiber, and simple sugars. 

## 5. Conclusions

Energy; carbohydrates, protein, and fat as percentages of energy; vitamin A; riboflavin; niacin; vitamin C; calcium; and iron were selected to be included in the nutritional standards of school lunches in Korea. The list of nutrients differs from the current standards in that it includes niacin, but not thiamine. It is also different in that the new list includes protein as a percentage of energy, while the current standards include protein not only as a percentage of energy, but also in absolute quantity. It is suggested that reference values be established for the selected nutrients through further research to revise the nutritional standards for school lunches in Korea.

## Figures and Tables

**Table 1:** Within-individual variance components ^1^ estimated through the Iowa State University (ISU) method.

Gender	Age (Years)	*n*	Energy (kcal)	Protein (g)	Carbohydrates (% Energy)	Protein (% Energy)	Fat (% Energy)	Vitamin A	Thiamin (mg)	Riboflavin (mg)	Niacin (mg NE ^3^)	Vitamin C (mg)	Calcium (mg)	Phosphorus (mg)	Sodium (mg)	Potassium (mg)	Iron (mg)
								(μg RAE ^2^)									
Male	**6–8**	**612**	**0.5949**	**0.6752**	**0.7494**	**0.7831**	**0.7507**	**0.7188**	**0.6970**	**0.6024**	**0.7071**	**0.7723**	**0.5355**	**0.5686**	**0.5997**	0.5752	0.6593
	9–11	668	0.6737	0.7237	0.9049	0.8609	0.9436	0.8145	0.8162	0.7129	0.7504	0.8419	0.6217	0.6205	0.7920	0.6526	0.6901
	12-14	634	0.7023	0.7318	0.8030	0.8716	0.8570	0.8210	0.8074	0.6769	0.7756	0.7506	0.6549	0.6600	0.7939	0.6303	0.7344
	15-17	491	0.5985	0.6491	0.8330	0.8828	0.8157	0.7417	0.7948	0.6451	0.6913	0.7503	0.6600	0.5779	0.6562	0.5924	0.6750
Female	6–8	584	0.6849	0.6989	0.8327	0.8638	0.8410	0.8374	0.7557	0.6759	0.7339	0.6887	0.6226	0.6231	0.7098	0.6356	0.6825
	9–11	601	0.6747	0.8032	0.9467	0.8411	0.9332	0.7574	0.7728	0.6851	0.8121	0.7073	0.6325	0.6809	0.6874	0.6089	0.6822
	12–14	553	0.6144	0.6851	0.8491	0.8866	0.8191	0.7308	0.7898	0.6589	0.7180	0.7048	0.6191	0.5935	0.7034	0.6342	0.6890
	15–17	467	0.6624	0.7704	0.9303	0.9907	0.8324	0.8613	0.7559	0.7058	0.7236	0.7309	0.6632	0.6572	0.7565	0.6377	0.7216
All		4610	0.5795	0.6467	0.8440	0.8727	0.8459	0.7702	0.7151	0.6426	0.6793	0.7408	0.6112	0.5744	0.6367	0.5909	0.6578

^1^ Calculated from the two-day, 24 h recall data provided by the 2007–2009 Dietary Intake Survey of Infants, Children, and Adolescents [24]. ^2^ Retinol activity equivalent. ^3^ Niacin equivalent.

**Table 2:** The percentage of the average usual energy intake to the estimated energy requirement (EER) among Korean school-aged children and adolescents.

Gender	Age (Years)	*n*	Average Usual Energy Intake (kcal)	EER (kcal)	Percentage of the Average Usual Energy Intake for the EER (%)
Male	6–8	399	1912	1700	112.5
	9–11	444	2169	2100	103.3
	12–14	407	2464	2500	98.6
	15–17	367	2592	2700	96.0
Female	6–8	383	1592	1500	106.1
	9–11	370	1942	1800	107.9
	12–14	373	1995	2000	99.8
	15–17	348	1922	2000	96.1

**Table 3:** The proportion of Korean school-aged children and adolescents with insufficient and excessive intake of the percentages of energy from carbohydrates, protein, and fat.

Gender	Age (Years)	*n*	Proportion of the Subjects below the AMDR ^1^ (%)			Proportion of the Subjects above the AMDR (%)		
			Carbohydrates	Protein	Fat	Carbohydrates	Protein	Fat
Male	6–8	399	16.5	0	1.8	27.1	0.5	12.5
	9–11	444	7.2	0	0.0	17.6	0.9	0.9
	12–14	407	17.0	0	1.0	21.4	0.5	5.9
	15–17	367	19.3	0	1.4	16.6	0.5	12.8
Female	6–8	383	3.9	0	1.8	36.6	0.0	3.4
	9–11	370	1.6	0	0.3	13.0	0.5	0.8
	12–14	373	8.6	0	1.9	22.8	0.5	8.8
	15–17	348	5.7	0	1.4	11.2	0.0	8.9
All		3091	10.1	0	1.2	20.1	0.5	6.6

^1^ Acceptable macronutrient distribution range; the AMDRs for carbohydrates, protein, and fat are 55%–65%, 7%–20%, and 15%–30%, respectively.

**Table 4:** The proportion of Korean school-aged children and adolescents with insufficient nutrient intake.

Gender	Age (Years)	*n*	Protein		Vitamin A		Thiamin		Riboflavin		Niacin		Vitamin C		Calcium		Phosphorus		Iron	
			EAR ^1^ (g)	% Below the EAR	EAR (μg RAE ^2^)	% Below the EAR	EAR (mg)	% Below the EAR	EAR (mg)	% Below the EAR	EAR (mg NE ^3^)	% Below the EAR	EAR (mg)	% Below the EAR	EAR (mg)	% Below the EAR	EAR (mg)	% Below the EAR	EAR (mg)	% Below the EAR
**Male**	**6–8**	399	25	0.0	320	47.1	0.6	0.0	0.7	3.8	7	2.5	40	21.3	580	64.2	490	0.8	7	4.5
	9–11	444	35	0.9	420	53.2	0.7	0.0	1.0	14.0	9	7.0	55	28.8	650	71.2	1000	37.4	8	3.6
	12–14	407	45	1.2	540	64.1	1.0	0.0	1.2	18.7	11	7.4	70	46.7	800	85.5	1000	0.2	11	10.1
	15–17	367	50	6.3	620	84.5	1.1	0.8	1.4	38.4	13	16.6	80	56.4	720	78.7	1000	27.2	11	13.6
Female	6–8	383	20	0.3	290	41.8	0.6	0.0	0.6	3.1	7	8.9	45	27.7	580	84.9	450	2.9	6	3.9
	9–11	370	30	0.3	380	43.6	0.7	0.0	0.8	7.0	9	9.7	60	44.6	650	74.1	1000	51.9	7	3.0
	12–14	373	40	5.9	470	72.7	0.9	1.1	1.0	27.6	11	28.7	75	60.3	740	90.3	1000	55.8	13	37.8 ^4^
	15–17	348	40	4.9	440	77.3	1.0	4.0	1.0	29.0	11	30.2	70	62.6	660	87.1	1000	60.6	11	41.1 ^4^
All		3091		2.4		60.0		0.7		17.3		13.4		42.8		79.2		28.9		14.1

^1^ Estimated average requirement. ^2^ Retinol activity equivalent. ^3^ Niacin equivalent. ^4^ The full probability approach was used to assess the iron intake for the females aged 12–17 years old.

**Table 5:** The proportion of Korean school-aged children and adolescents with excessive nutrient intake.

	Age (Years)	*n*	Vitamin A		Niacin		Vitamin C		Calcium		Phosphorus		Sodium		Iron	
			UL ^1^ (μg RAE)	% Above the UL	UL (mg NE ^2^)	% Above the UL	UL (mg)	% Above the μL	UL (mg)	% Above the UL	UL (mg)	% Above the UL	Intake Goal ^3^ (mg)	% Above the Intake Goal	UL (mg)	% Above the UL
Male	6–8	399	1000	6.8	350	0	700	0	2500	0	3000	0		*n*/a ^4^	40	0.3
	9–11	444	1500	0.9	500	0	1000	0	3000	0	3500	0	2000	93.2	40	0.5
	12–14	407	2100	2	700	0	1400	0	3000	0	3500	6.6	2000	98.3	40	7.4
	15–17	367	2300	0	800	0	1500	0	3000	0	3500	0	2000	94.6	45	2.7
Female	6–8	383	1000	2.9	350	0	700	0	2500	0	3000	0		n/a ^4^	40	0
	9–11	370	1500	3	500	0	1000	0	3000	0.3	3500	1.4	2000	81.4	40	0
	12–14	373	2100	0.8	700	0	1400	0	3000	0	3500	0	2000	85.8	40	0
	15–17	348	2300	0	800	0	1500	0	3000	0	3500	0	2000	86.5	45	0.3
All		3,091		2.1		0		0		0		1.0		90.2		1.4

^1^ Tolerable upper intake level. ^2^ Niacin equivalent. ^3^ For sodium, the value is the proportion above the intake goal of 2000 mg. ^4^ In the 2015 Dietary Reference Intakes for Koreans, there are no intake goals for males and females aged 6–8 years old; the risk of excess intake could not be assessed.

**Table 6:** The probability of inadequate iron intake for Korean females aged 12–17 years old, calculated using the full probability approach.

Probability of Inadequacy (%) (a)	Female 12–14 Years (*n* = 373)			Female 15–17 Years (*n* = 348)		
	Range of the Intake with the Probability of Inadequacy ^1^ (mg/day, 12% Bioavailability)	Proportion of Females in this Intake Range ^2^ (%) (b)	Prevalence of Inadequacy ^3^ (%) (a × b)	Range of the Intake with the Probability of Inadequacy ^1^ (mg/day, 12% Bioavailability)	Proportion of Females in this Intake Range ^2^ (%) (c)	Prevalence of inadequacy ^3^ (%) (a × c)
0	>20.9	8.3	0.00	>21.6	10.6	0.00
0.04	18.5–20.9	4.8	0.19	19.2–21.6	5.2	0.21
0.08	16.2–18.5	9.1	0.73	16.8–19.2	10.6	0.85
0.15	14.0–16.2	16.9	2.54	14.7–16.8	12.1	1.82
0.25	12.7–14.0	8.8	2.20	13.4–14.7	6.9	1.73
0.35	11.7–12.7	11.5	4.03	12.3–13.4	10.6	3.71
0.45	10.9–11.7	7	3.15	11.6–12.3	6	2.70
0.55	10.2–10.9	7	3.85	10.8–11.6	4.9	2.70
0.65	9.3–10.2	8	5.20	10.0–10.8	7.5	4.88
0.75	8.6–9.3	5.1	3.83	9.3–10.0	4	3.00
0.85	7.5–8.6	7	5.95	8.2–9.3	10.3	8.76
0.93	6.7–7.5	2.9	2.70	7.3–8.2	4.6	4.28
0.96	6.1–6.7	1.6	1.54	6.8–7.3	2	1.92
1	<6.1	1.9	1.90	<6.8	4.6	4.60
Total probability of inadequate intake for females ^4^ (%)	−	−	37.79	−	−	41.13

^1^ The intake ranges for the probability of inadequate iron intake of females aged 11–13 and 14–18 years old with 12% bioavailability [27] were applied to females aged 12–14 and 15–17 years old, respectively. ^2^ The proportion of females in this intake range in the estimated usual intake. ^3^ The probability of inadequacy multiplied by the proportion of females in this intake range. ^4^ The sum of the prevalence of inadequacy.

## Supplementary Materials

The following are available online at https://www.mdpi.com/2072-6643/11/11/2578/s1, Table S1: Korean school-aged children and adolescents’ usual intake of energy and protein; Table S2: Korean school-aged children and adolescents’ usual intake of the percentages of energy from carbohydrates, protein, and fat; Table S3: Korean school-aged children and adolescents’ usual intake of vitamin A, thiamin, and riboflavin; Table S4: Korean school-aged children and adolescents’ usual intake of niacin and vitamin C; Table S5: Korean school-aged children and adolescents’ usual intake of calcium and phosphorus; Table S6: Korean school-aged children and adolescents’ usual intake of sodium, potassium, and iron.
