# Supplementary material for: Selection of Target Nutrients for the Nutritional Standards of School Lunches in Korea
Source: Nutrients. 2019 Oct 25;11(11):2578. doi: 10.3390/nu11112578 (PMC6893548; doi:10.3390/nu11112578)
Supplement: Supplementary file 1 [file nutrients-11-02578-s001.pdf]

Table S1. Korean school-aged children and adolescents' usual intake of energy and protein

	Gender	Age (years)	n (3,091)	m	SD	Percentiles						
						5th	10th	25th	50th	75th	90th	95th
Energy (kcal)	Male	6–8	399	1,912.2	520.7	1,223.1	1,325.7	1,561.6	1,845.9	2,162.8	2,567.1	2,843.5
		9–11	444	2,169.2	566.5	1,382.7	1,519.3	1,774.4	2,089.1	2,501.8	2,870.5	3,179.6
		12–14	407	2,463.8	591.6	1,563.5	1,762.1	2,033.0	2,417.1	2,793.0	3,314.4	3,487.7
		15–17	367	2,592.1	817.6	1,486.4	1,650.9	2,010.8	2,478.3	3,041.9	3,683.7	4,136.5
	Female	6–8	383	1,592.2	336.9	1,073.9	1,172.5	1,360.6	1,556.1	1,815.6	2,039.0	2,224.8
		9–11	370	1,942.1	506.1	1,232.8	1,358.1	1,581.6	1,854.0	2,255.7	2,649.4	2,867.5
		12–14	373	1,995.3	632.5	1,174.8	1,332.9	1,581.0	1,890.9	2,291.0	2,820.1	3,268.8
		15–17	348	1,922.2	569.0	1,120.9	1,235.4	1,525.9	1,857.0	2,282.9	2,663.2	2,938.0
Protein (g)	Male	6–8	399	66.4	20.0	40.5	44.7	52.4	62.9	77.0	91.8	104.5
		9–11	444	78.0	25.1	45.5	50.4	60.5	75.0	90.3	112.0	122.0
		12–14	407	90.4	28.3	51.5	57.7	71.4	86.5	105.7	125.7	139.8
		15–17	367	96.8	39.6	48.5	54.6	69.9	89.9	116.0	147.6	167.8
	Female	6–8	383	52.9	13.7	32.0	36.2	43.6	51.4	61.3	70.5	76.5
		9–11	370	72.3	24.8	41.3	46.5	55.5	66.4	84.2	105.8	117.5
		12–14	373	73.1	32.6	38.6	43.6	53.0	65.3	84.4	109.0	134.8
		15–17	348	68.4	21.5	40.0	44.7	53.5	64.3	80.2	97.4	108.4

Table S2. Korean school-aged children and adolescents' usual intake of the percentages of energy from carbohydrates, protein, and fat

	Gender	Age (years)	n (3,091)	m	SD	Percentiles						
						5th	10th	25th	50th	75th	90th	95th
Carbohydrates (% energy)	Male	6–8	399	61.1	5.8	51.0	53.4	57.4	61.6	65.6	68.4	70.0
		9–11	444	61.2	4.0	54.2	55.8	58.7	61.4	63.8	66.2	67.9
		12–14	407	60.4	5.7	50.2	52.8	56.9	60.4	64.4	67.3	69.5
		15–17	367	59.5	5.6	49.7	51.9	56.1	59.8	63.2	66.7	68.8
	Female	6–8	383	63.6	4.7	55.5	57.3	60.5	63.4	67.0	69.3	71.6
		9–11	370	61.8	3.0	56.9	57.9	59.9	61.8	63.9	65.4	66.5
		12–14	373	61.4	4.9	53.9	55.3	58.1	61.4	64.8	67.6	69.0
		15–17	348	60.6	3.5	54.8	56.1	58.5	60.6	63.0	65.2	66.4
Protein (% energy)	Male	6–8	399	13.9	1.9	11.1	11.7	12.5	13.7	15.2	16.6	17.2
		9–11	444	14.2	1.9	11.4	11.9	12.9	14.1	15.3	16.6	17.7
		12–14	407	14.4	1.9	11.6	12.1	13.1	14.3	15.4	17.0	17.8
		15–17	367	14.5	1.8	11.8	12.2	13.2	14.4	15.5	17.0	17.5
	Female	6–8	383	13.3	1.6	10.9	11.3	12.2	13.2	14.3	15.4	16.1
		9–11	370	13.9	1.9	11.2	11.6	12.6	13.8	15.0	16.4	17.2
		12–14	373	13.9	1.7	11.4	11.8	12.8	13.7	15.0	16.2	16.9
		15–17	348	14.2	0.6	13.3	13.5	13.8	14.2	14.5	14.9	15.1
Fat (% energy)	Male	6–8	399	24.1	4.9	16.1	17.8	20.8	23.9	27.2	30.8	32.8
		9–11	444	23.5	2.5	19.5	20.2	21.8	23.4	25.2	26.8	27.6
		12–14	407	23.8	4.0	17.2	18.9	21.1	23.8	26.5	29.3	30.4
		15–17	367	24.6	4.8	16.9	18.1	21.2	24.4	27.6	30.7	32.8
	Female	6–8	383	22.5	3.9	16.4	17.6	19.9	22.6	25.1	27.6	29.5
		9–11	370	23.6	2.6	19.5	20.3	21.9	23.5	25.3	26.9	27.9
		12–14	373	23.9	4.3	17.1	18.7	20.9	24.2	26.6	29.7	31.1
		15–17	348	24.0	4.3	16.9	18.8	21.0	23.9	26.6	29.8	31.5

Table S3. Korean school-aged children and adolescents' usual intake of vitamin A, thiamin, and riboflavin

	Gender	Age (years)	n (3,091)	m	SD	Percentiles						
						5th	10th	25th	50th	75th	90th	95th
Vitamin A (㎍ RAE)	Male	6–8	399	456.0	470.4	156.0	178.7	240.0	327.7	488.8	825.2	1,085.7
		9–11	444	473.7	275.4	186.7	220.7	281.2	397.1	575.6	864.3	985.4
		12–14	407	564.9	484.5	186.5	221.0	292.2	422.2	682.3	1,010.8	1,321.7
		15–17	367	423.2	230.8	169.5	199.4	263.6	369.4	532.1	737.6	843.6
	Female	6–8	383	389.9	267.8	165.1	186.9	234.3	316.9	455.9	670.3	799.4
		9–11	370	548.9	409.0	167.3	200.7	257.5	440.1	679.6	1,051.4	1,313.6
		12–14	373	442.1	390.9	136.9	160.0	221.9	330.3	506.3	906.0	1,162.4
		15–17	348	353.5	144.5	179.8	203.0	250.9	324.6	427.3	548.0	617.2
Thiamin (㎎)	Male	6–8	399	1.74	0.52	1.03	1.16	1.38	1.67	2.01	2.38	2.68
		9–11	444	1.98	0.47	1.30	1.40	1.62	1.94	2.25	2.59	2.84
		12–14	407	2.29	0.61	1.46	1.59	1.85	2.20	2.61	3.09	3.40
		15–17	367	2.45	0.73	1.39	1.61	1.95	2.32	2.88	3.42	3.78
	Female	6–8	383	1.43	0.35	0.92	0.99	1.18	1.39	1.65	1.85	2.08
		9–11	370	1.77	0.48	1.07	1.24	1.40	1.69	2.03	2.43	2.64
		12–14	373	1.80	0.50	1.14	1.24	1.45	1.71	2.07	2.45	2.70
		15–17	348	1.79	0.57	1.01	1.12	1.41	1.70	2.09	2.54	2.80
Riboflavin (㎎)	Male	6–8	399	1.37	0.47	0.75	0.83	1.06	1.28	1.63	1.97	2.27
		9–11	444	1.54	0.51	0.86	0.93	1.18	1.46	1.85	2.22	2.42
		12–14	407	1.72	0.60	0.90	1.05	1.29	1.63	2.09	2.46	2.74
		15–17	367	1.65	0.64	0.76	0.91	1.19	1.55	2.01	2.47	2.81
	Female	6–8	383	1.12	0.33	0.63	0.74	0.87	1.09	1.32	1.58	1.68
		9–11	370	1.41	0.49	0.76	0.86	1.08	1.33	1.68	2.05	2.28
		12–14	373	1.36	0.56	0.71	0.79	0.97	1.25	1.60	2.03	2.39
		15–17	348	1.28	0.45	0.64	0.76	0.97	1.20	1.53	1.86	2.16

Table S4. Korean school-aged children and adolescents' usual intake of niacin and vitamin C

	Gender	Age (years)	n (3,091)	m	SD	Percentiles						
						5th	10th	25th	50th	75th	90th	95th
Niacin (㎎ NE)	Male	6–8	399	13.4	4.7	7.4	8.6	10.0	12.7	15.7	19.4	22.0
		9–11	444	15.3	5.0	8.6	9.7	11.8	14.6	18.1	22.0	25.3
		12–14	407	18.0	5.4	10.4	11.8	14.4	17.2	20.8	25.5	28.8
		15–17	367	19.7	7.4	9.6	11.3	14.7	18.3	23.6	29.8	33.6
	Female	6–8	383	10.8	3.1	6.2	7.2	8.6	10.4	12.9	14.9	16.5
		9–11	370	13.6	4.0	8.2	9.0	10.6	13.1	15.9	19.0	20.9
		12–14	373	14.1	5.0	7.7	8.8	10.8	13.1	16.8	19.9	23.2
		15–17	348	13.9	4.9	7.2	8.3	10.2	13.1	16.3	20.7	22.9
Vitamin C (㎎)	Male	6–8	399	67.4	37.5	23.7	29.5	42.5	57.8	87.2	114.7	131.0
		9–11	444	77.7	35.7	32.3	37.0	51.3	72.4	97.2	126.9	139.1
		12–14	407	86.4	56.9	23.2	30.6	47.1	73.6	108.7	161.6	198.3
		15–17	367	91.9	75.1	23.0	28.9	45.7	70.4	113.5	183.9	238.4
	Female	6–8	383	83.0	58.2	21.0	27.0	42.7	66.8	110.2	159.0	208.0
		9–11	370	83.9	63.0	23.2	27.6	42.6	66.0	108.7	155.1	188.6
		12–14	373	76.3	51.1	20.9	26.5	42.4	64.2	99.8	143.2	170.5
		15–17	348	67.7	45.4	19.3	24.0	36.4	56.7	87.1	128.9	153.6

Table S5. Korean school-aged children and adolescents' usual intake of calcium and phosphorus

	Gender	Age (years)	n (3,091)	m	SD	Percentiles						
						5th	10th	25th	50th	75th	90th	95th
Calcium (㎎)	Male	6–8	399	543.7	255.5	221.9	280.3	374.4	494.6	650.6	858.6	993.3
		9–11	444	554.5	214.9	261.7	299.9	405.6	519.6	688.9	848.2	969.2
		12–14	407	577.1	237.4	265.0	308.6	419.3	543.7	699.8	875.8	1005.1
		15–17	367	565.7	269.9	244.7	293.4	373.5	521.0	681.9	908.5	1051.7
	Female	6–8	383	423.1	160.8	195.5	232.8	303.9	403.5	526.8	626.1	702.3
		9–11	370	562.5	338.5	243.5	277.5	366.2	488.3	656.8	917.3	1218.9
		12–14	373	466.4	207.2	214.1	246.3	322.9	425.2	562.2	737.8	903.8
		15–17	348	434.5	173.3	192.0	225.3	313.1	418.0	536.3	687.3	745.0
Phosphorus (㎎)	Male	6–8	399	1,040.5	323.2	617.2	671.3	812.5	1,010.3	1,185.7	1,452.8	1,596.4
		9–11	444	1,137.9	335.5	639.8	749.9	905.7	1,104.5	1,321.4	1,603.6	1,767.7
		12–14	407	2,463.7	622.0	1,526.6	1,732.9	2,012.2	2,408.7	2,804.7	3,361.0	3,546.4
		15–17	367	1,289.0	463.1	633.4	725.9	966.2	1,221.2	1,538.7	1,889.8	2,130.6
	Female	6–8	383	828.4	215.9	492.2	568.6	687.6	807.3	969.4	1,127.6	1,209.2
		9–11	370	1,167.0	689.8	615.0	690.6	814.3	979.1	1,282.7	1,714.3	2,620.7
		12–14	373	1,004.8	343.1	554.7	630.8	776.3	942.5	1,178.6	1,410.9	1,627.1
		15–17	348	973.3	325.8	508.0	593.4	747.7	927.3	1,157.7	1,408.8	1,545.8

Table S6. Korean school-aged children and adolescents' usual intake of sodium, potassium, and iron

	Gender	Age (years)	n (3,091)	m	SD	Percentiles						
						5th	10th	25th	50th	75th	90th	95th
Sodium (㎎)	Male	6–8	399	2,744.7	1,106.9	1,284.1	1,582.7	1,954.4	2,591.9	3,269.6	4,123.2	4,812.4
		9–11	444	3,343.2	1,024.7	1,948.0	2,151.2	2,632.7	3,208.2	3,879.4	4,731.3	5,199.4
		12–14	407	3,997.6	1,268.7	2,360.9	2,576.3	3,139.2	3,778.1	4,671.6	5,610.4	6,332.5
		15–17	367	4,373.9	1,812.3	1,947.8	2,377.2	3,024.6	4,135.1	5,264.5	6,894.6	7,678.2
	Female	6–8	383	2,295.2	753.2	1,324.0	1,483.9	1,775.0	2,150.3	2,677.8	3,221.1	3,597.5
		9–11	370	3,029.0	1,255.7	1,505.5	1,738.6	2,154.7	2,790.8	3,677.9	4,600.0	5,262.2
		12–14	373	3,163.0	1,309.3	1,641.2	1,843.8	2,279.4	2,924.6	3,692.1	4,834.8	5,749.4
		15–17	348	3,157.2	1,160.7	1,661.5	1,913.6	2,315.0	3,010.3	3,752.3	4,575.9	5,583.9
Potassium (㎎)	Male	6–8	399	2,414.3	815.7	1,376.3	1,557.2	1,861.2	2,292.8	2,792.4	3,497.5	3,846.5
		9–11	444	2,654.2	818.7	1,488.2	1,727.0	2,046.8	2,544.9	3,173.5	3,713.0	4,224.2
		12–14	407	3,080.5	1,187.0	1,568.5	1,826.6	2,276.5	2,827.3	3,687.9	4,621.5	5,100.3
		15–17	367	3,018.1	1,122.6	1,449.9	1,730.5	2,220.0	2,908.0	3,604.3	4,534.9	5,140.4
	Female	6–8	383	2,102.2	657.2	1,196.9	1,372.5	1,610.4	2,042.6	2,467.5	2,953.4	3,313.7
		9–11	370	2,475.2	760.8	1,403.6	1,629.3	1,924.2	2,385.4	2,933.1	3,531.7	3,857.8
		12–14	373	2,428.4	872.0	1,274.8	1,469.9	1,830.0	2,306.6	2,843.0	3,544.7	4,099.3
		15–17	348	2,341.8	842.1	1,196.2	1,418.3	1,755.9	2,197.4	2,842.3	3,410.4	3,889.1
Iron (㎎)	Male	6–8	399	13.0	5.0	7.3	8.2	9.8	11.9	15.2	19.1	21.4
		9–11	444	15.3	5.8	8.3	9.1	11.2	14.0	18.2	22.7	26.0
		12–14	407	20.9	17.4	9.7	11.0	13.0	16.7	22.7	33.3	49.1
		15–17	367	19.3	8.9	9.0	10.0	12.9	17.8	23.2	30.1	35.6
	Female	6–8	383	11.5	4.6	6.2	7.0	8.5	10.6	13.5	16.9	19.1
		9–11	370	13.6	5.1	7.5	8.4	10.2	12.7	15.6	19.4	22.5
		12–14	373	13.5	5.3	7.2	8.0	9.9	12.5	15.8	20.2	23.7
		15–17	348	14.4	6.6	6.8	8.0	9.9	12.9	17.3	21.8	27.0
